# Evaluation of the Antioxidant and antimicrobial Properties of Dorema aucheri plant

**Published:** 2012-10-30

**Authors:** M Khanahmadi, S Sh Miraghaee, I Karimi

**Affiliations:** 1 Department of Chemistry, Kermanshah Branch of ACECR, Kermanshah, Iran; 2 Department of pharmachognosy and biotechnology. School of pharmacy, Kermanshah University of medicinal science, Kermanshah, Iran; 3 Professor college of Veterinary medicine Razi University, Tehran, Iran

**Keywords:** Dorema aucheri, antioxidant property, antimicrobial property

Dear Editor,

Seven species have been reported in the genus Dorema, family Umbelliferae, in the flora of Iran, among which two are endemic: D. aucheri Boiss. and D. ammoniacum D. Don (1). In Iranian traditional medicine, Dorema aucheri has been employed as stimulant, nervonic, antispasmodic, bronchodilator, expectorant, kidney stone repellent, emmenagogue and analgesic for visceral pain. This plant eaten as a green by some people in west part of Iran. D. aucheri in microliter scale to mice caused getting cancer dramatically. There are several reports on chemical composition, antihyperlipidemic and antihypercholesterolamic effects of this plant (2). Previous phytochemical investigations on this species revealed the presence of exudates flavonoides (3).

Although thorough phytochemical and some evidence-based pharmacological studies have been carried out on the trunk and resin of D. aucheri, little is known about the antibacterial and antioxidant effects of ethanol extract of D. aucheri. The purpose of the present study was to elucidate these effects in vitro.

For this purpose Dorema aucheri Boiss. was collected from Oramanat area at Kermanshah province, west Iran (34°18′N, 47°3′E and 1420 m above sea level) in May 2010 and identified in Kermanshah Research Institute of Forests and Rangelands. ethanolic extract and essential oil of dried powdered aerial parts of plant were prepared by us. Essential oil components were investigated by Gc/Mass. Total phenolic compounds in extracts were determined spectrophotometricaly using the Folin-Ciocalteu reagent. We use DPPH and FTC method for evaluation of Antioxidant activity assessed by using. 

**Figure 1:**
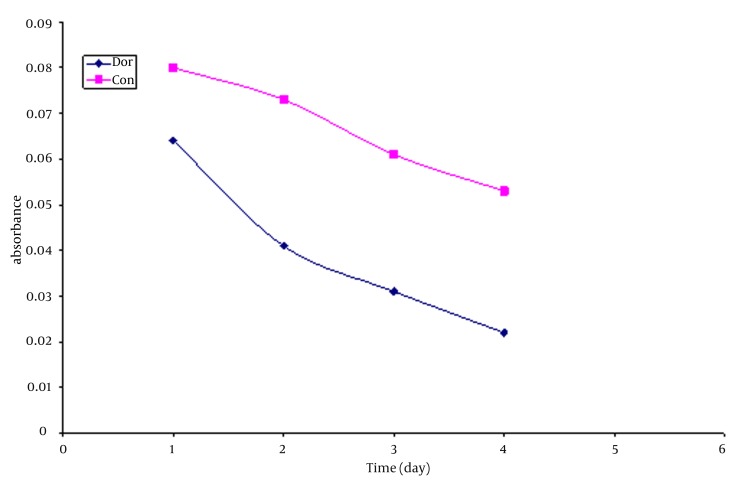
Results showed this plant could be candidates for some of its biological activities and therefore for its therapeutic use

Antibacterial effects of ethanolic extract of this plant was analyzed by disc diffusion and microdilution Broth methods (4).

thirty six compounds were identified in the leaf oil of the plant, representing about 99.86% of the total oil with Curzerene (18.7%), Spathulenil (6.68%) and Isohibaene (6.16%) and Gemberen (6.66%) as the major constituents. (table.1) extract exhibited a notable dose dependent inhibition of the DPPH activity, with a 50% inhibition (IC50) at a concentration of 0.2 mg/ml (5). We use Folin – ciocaltue method for determination of total phenolic content in the ethanol extracts of D. aucheri (5). (Fig1) The content of phenolic compounds was 38.42 mg.

Plant extracts and plant-derived antioxidant compounds potentiate body’s antioxidant defense, they are antioxidants of choice because of their lower toxicity and side effects over the synthetic ones. Also, they are relatively cheaper and are easily accessible. The extract of D. aucheri has shown to possess in vitro antioxidant and antibacterial activities. The reducing ability of a compound generally depends on the presence of reductions, which exhibit antioxidant activity by breaking the free radical chain though donation of a hydrogen atom. Further studies need for the isolation and characterization of the active principles component for these activities in this plant.

**Table 1:** Main Components of Dorema aucheri essential oil identified by Gc and GC/MS

NO	RT	%	Name	KI
1	54.99	3.98	b-Guryunene	1498.1
2	44.66	18.37	Curzerene	1516
3	48	6.68	Spathulenol	1600
4	49.94	7.72	a- Eudesmol	1650
5	53.34	7.58	Valeranone	1749.2
6	58.59	6.169	Isohibaene	1891
7	59.85	6.66	Gembrene	1929
	99.86		Sum
